# Identification of Amino Acids That Regulate Angiogenesis and Alter Pathogenesis of a Mouse Model of Choroidal Neovascularization

**DOI:** 10.3390/nu17183006

**Published:** 2025-09-19

**Authors:** Chenchen Li, Jiawen Wu, Yingke Zhao, Jing Zhu, Xinyu Zhu, Yan Chen, Jihong Wu

**Affiliations:** 1 Department of Opthalmology, Eye and ENT Hospital, Fudan University, Shanghai 200031, China; lichenchen@fudan.edu.cn (C.L.); 20211260003@fudan.edu.cn (J.W.); ykzhao@fudan.edu.cn (Y.Z.); 2 Shanghai Institute of Nutrition and Health, University of Chinese Academy of Sciences, Chinese Academy of Sciences, Shanghai 200031, China; zhujing2020@sinh.ac.cn (J.Z.); zhuxy2@shanghaitech.edu.cn (X.Z.); 3 Shanghai Key Laboratory of Visual Impairment and Restoration, Science and Technology Commission of Shanghai Municipality, Shanghai 200031, China; 4 Key Laboratory of Myopia and Related Eye Diseases, NHC, Shanghai 200031, China; 5 Key Laboratory of Myopia and Related Eye Diseases, Chinese Academy of Medical Sciences, Shanghai 200031, China

**Keywords:** essential amino acid restriction, heme-regulated inhibitor, general control nonderepressible 2, activating transcription factor 4, angiogenesis, choroidal neovascularization

## Abstract

**Background**: Metabolic stress from amino acid (AA) insufficiency is increasingly linked to pathological angiogenesis, but specific essential AA (EAA) roles remain undefined. Neovascular age-related macular degeneration (AMD), a major cause of blindness driven by aberrant ocular neovascularization, has limited efficacy with current VEGFA-targeting therapies. We sought to identify specific EAAs that regulate pathological angiogenesis and dissect their mechanisms to propose new therapeutic strategies. **Methods**: Human retinal microvascular endothelial cells (HRMVECs) were used to identify angiogenesis-regulating amino acids through systematic EAA screening. The molecular mechanism was investigated using shRNA-mediated knockdown of key stress response regulators (HRI, PKR, PERK, GCN2) and ATF4. Angiogenesis was assessed via tubule formation and migration assays. Therapeutic potential was examined in a laser-induced choroidal neovascularization (CNV) mouse model, evaluated by fluorescein angiography and histomorphometry. **Results**: Deprivation of methionine, lysine, and threonine potently induced capillary-like tube formation (*p* < 0.01). Mechanistically, restriction of these three EAAs activated HRI and GCN2 kinases, converging on eIF2α phosphorylation to induce ATF4 and its target VEGFA. Dual, but not single, knockdown of HRI and GCN2 abolished eIF2α-ATF4 signaling and angiogenic responses. Restricting these EAAs exacerbated CNV area in mice. **Conclusions**: Our findings reveal a coordinated HRI/GCN2-ATF4-VEGFA axis linking EAA scarcity to vascular remodeling, establishing proof-of-concept for targeting this pathway in CNV. This work highlights the therapeutic potential of modulating specific AA availability or targeting the HRI/GCN2-ATF4 axis to treat CNV.

## 1. Introduction

Recent studies highlight the pivotal role of amino acid metabolism in maintaining blood vessel stability and influencing eye-related diseases. Although amino acids that the body can produce, such as arginine and glutamine, are known to contribute to blood vessel growth driven by VEGF [1,2], the ways in which indispensable amino acids reshape metabolism in eye blood vessel disorders are still not fully understood. This knowledge gap persists despite established links between EAA deprivation and compromised antioxidant defense mechanisms—particularly through glutathione (GSH) depletion in the gamma-glutamyl cycle [3]-which exacerbates oxidative retinal damage and pathological angiogenesis in age-related macular degeneration [4]. Intriguingly, mitochondrial oxidative phosphorylation (OXPHOS) perturbations generate reactive oxygen species (ROS) that activate the integrated stress response via HRI kinase, triggering eukaryotic translation initiation factor 2α (eIF2α) phosphorylation and subsequent activating transcription factor 4 (ATF4)-mediated transcriptional programs [5,6,7]. Paradoxically, while heme-regulated inhibitor (HRI) activation suppresses angiogenesis in extraocular contexts [8,9], its interplay with nutrient-sensing pathways in retinal vascular pathologies remains unexplored.

Existing models fall short in clarifying how overall essential amino acid levels interact with local cellular pressures to promote abnormal blood vessel growth in the choroid, a key feature of the wet form of age-related macular degeneration [2,10,11]. Important unanswered issues include: (1) Which particular essential amino acids control the ability of retinal blood vessel cells to form new vessels, (2) How sensors for amino acid shortages (like HRI and GCN2) communicate with factors that encourage vessel growth, and (3) Whether adjusting metabolic pathways could offer new treatments to slow down AMD. Addressing these questions requires a systematic dissection of EAA-mediated regulatory networks in human retinal microvasculature.

Our investigation reveals methionine, lysine, and threonine deprivation as potent drivers of pathological angiogenesis through an unprecedented convergence mechanism. High-resolution screening in HRMVECs demonstrates that these EAAs coordinately regulate eIF2α phosphorylation dynamics via HRI and GCN2 kinases, establishing a bimodal control node for ATF4 activation. This transcription factor subsequently amplifies VEGFA expression through direct promoter engagement, bypassing canonical hypoxia-responsive pathways. Crucially, dietary restriction of these EAAs in a murine CNV model paradoxically enhanced neovascular lesion formation, suggesting their dual role as metabolic substrates and signaling regulators. The principal aims of this study were therefore: (1) to systematically identify which specific EAAs govern pathological angiogenesis in human retinal endothelial cells; (2) to dissect the underlying molecular mechanism, with a focus on the potential interplay between nutrient-sensing kinases (HRI/GCN2) and the downstream eIF2α-ATF4-VEGFA axis; and (3) to validate the pathophysiological relevance of these findings using a laser-induced choroidal neovascularization mouse model, a well-established in vivo system that recapitulates key features of human neovascular AMD and is highly relevant for testing metabolic and dietary interventions. This approach provides a critical translational bridge between cellular nutrient sensing and ocular vascular pathology. These findings redefine our understanding of nutrient stress adaptation in retinal diseases and propose targeted modulation of specific AA availability or downstream stress effectors as a potential therapeutic strategy against AMD-associated vascular pathology.

## 2. Materials and Methods

### 2.1. Mice

All animal experiments were conducted following the guidelines of the Institutional Animal Care and Use Committee of the Shanghai Institute of Nutrition and Health, Chinese Academy of Sciences. 8-week male C57BL/6 mice purchased from SLAC (Shanghai, China) were used for all experiments, Except where indicated, animals were maintained under pathogen-free conditions with ad libitum (AL) access to food and water, 12-h light/12-h dark cycles, temperature between 20 and 23 °C with 30–70% relative humidity at the animal facility of Shanghai Institute for Biological Sciences (SIBS), Chinese Academy of Sciences (CAS) with approval number SINH-2024-CY-1 (approval date 30 May 2024).

Experimental diets were based on FBSH Biotechnology diets A0021B with approximately 18% of calories from protein, 12% from fat, and 71% from carbohydrate. The methionine (M)-, lysine (K)-, and threonine (T)-restricted diets—containing 1.5 g/kg M, 3.5 g/kg K, and 2 g/kg T, respectively—were formulated as isocaloric diets with 17% protein and 71% carbohydrate content and provided AL to respective experimental groups. The Experimental diets were purchased from FBSH Biotechnology Co., Ltd. (Shanghai, China). The nutrition information of all the diets used in this study is given in Table S1.

A laser-induced CNV mouse model was developed using well-established experimental methods. Mice were initially anesthetized with zoletil 50 (Virbac, Carros, France), and their pupils were dilated using tropicamide (Santen Pharmaceutica, Osaka, Japan). Medical ultrasonic couplant was applied after full pupil dilation, which occurred within 3 to 5 min, to maintain corneal moisture. Each eye underwent three rounds of laser photocoagulation around the optic nerve while avoiding major retinal blood vessels (532 nm wavelength, 110 mW power, 150 ms duration, 300 μm fixed spot size). The formation of bubbles during laser photocoagulation, indicating Bruch membrane rupture, confirmed the procedure’s effectiveness.

### 2.2. Cell Lines and Culture Conditions

Human retinal microvascular endothelial cells were purchased from QiDa (CD5091, Shanghai QiDa Biotechnology, Shanghai, China) and used between passages 1 and 15. HRMVECs were cultured in Endothelial cell medium (ECM) purchased from ScienCell (1001, ScienCell, Carlsbad, CA, USA) supplemented with 5% fetal bovine serum (FBS) and endothelial cell growth supplement (ECGS, Cat. No. 1052) at 37 °C in a humidified incubator at 5% CO_2_.

Essential amino acid restriction mediums were based on human umbilical vein endothelial cell growth medium (PWL121, MeilunBio, Dalian, China) supplemented with 10% Dialyzed FBS (FBS-DU075, Serana, Brandenburg, Germany), endothelial cell growth supplement, and heparin.

### 2.3. Gene Expression Analysis by Quantitative PCR (qPCR)

Total RNA was isolated with Ambion Trizol reagent according to the manufacturer’s instructions. Total RNA was reverse transcribed using the FastQuant RT kit (Tiangen Biotech, Beijing, China). Real-time PCR was carried out in an ABI 7900HT Fast Real-Time PCR System (AB Applied Biosystems, Warrington, UK) with SYBR Green PCR master mix (AB Applied Biosystems). The primers were as follows: 5′-AGCTGCGCTGATAGACATCC-3′ and 5′-CTACCTCCACCATGCCAAGT-3′ for human Vegfa, 5′-CTATACCCAACAGGGCATCC-3′, and 5′-GTCCCTCCAACAACAGCAAG-3′ for human atf4, 5′-ACCCCGAATATGACGAATCTGA-3′ and 5′-CAAGTGCTCCAGCAAAGAAAC-3′ for human Hri, 5′-GCCGCTAAACTTGCATATCTTCA-3′ and 5′-TCACACGTAGTAGCAAAAGAACC-3′ for human Pkr, 5′-ACGATGAGACAGAGTTGCGAC-3′ and 5′-ATCCAAGGCAGCAATTCTCCC-3′ for human Perk, 5′-TCTTTGAACTGGCTTACCACG-3′ and 5′-GCAGGATTTCACGTTGCTCC-3′ for human Gcn2. The relative changes in gene expression were normalized against Actin mRNA expression.

### 2.4. Immunoblotting

Cells were homogenized with lysis buffer (Beyotime, Shanghai, China), normalized for protein content, boiled with SDS loading buffer, and separated by SDS-PAGE. Proteins were transferred to PVDF membrane (Sigma-Aldrich, St. Louis, MO, USA) and blotted for ATF4 (11815, Cell Signaling Technology, Danvers, MA, USA), HIF1a (36169, Cell Signaling Technology), p-eIF2a (9712, Cell Signaling Technology), total eIF2a (9722, Cell Signaling Technology), HRI (A3415, ABclonal, Woburn, MA, USA), total PKR (A19545, ABclona), p-PKR (AP1134, ABclonal), total PERK (A188196, ABclonal), p-PERK (AP1501, ABclonal), total GCN2 (3302, Cell Signaling Technology), p-GCN2 (AP1356, ABclonal), p-AKT (9271, Cell Signaling Technology), total AKT (9272, Cell Signaling Technology), p-mTORC1 (2971, Cell Signaling Technology), total mTORC1 (2972, Cell Signaling Technology), p-P70 S6k (9204, Cell Signaling Technology), total P70 S6k (9202, Cell Signaling Technology), p-4E-BP1 (9451, Cell Signaling Technology), total 4E-BP1 (9452, Cell Signaling Technology), and Actin (4970, Cell Signaling Technology) and secondarily with HRP-conjugated anti-rabbit antibody (Cell Signaling Technology). The images were captured on the visualization instrument Tanon-5200 (Tanon, Shanghai, China).

### 2.5. VEGF ELISAs

The cell supernatant was centrifuged at 4000 r/min for 3 min, and the resulting supernatant was collected for further analysis. Human VEGF ELISA kits (Lanso, catalog number AE98006, North Vancouver, BC, Canada) were used to perform assays according to the manufacturer’s instructions, with each analysis conducted on 10 μL of cell culture supernatant.

### 2.6. Cell Viability Assay

HRMVECs were seeded in 96-well culture plates (2.0 × 10^3^ cells/well) and grown overnight. After treatment with different essential amino acid-restricted human umbilical vein endothelial cell growth medium supplemented with 10% Dialyzed FBS, endothelial cell growth supplement, and heparin for 24 h, the cells were incubated with CCK-8 (MA0218, Meilunbio) solution (10 μL/well) and cultured at 37 °C for another 1 h. The absorbance of the dissolved solutions was detected at 450 nm on a microplate reader (AMR-100, Aosheng, Ningbo, China). The cell viability rate was calculated as follows: (absorbance of amino acid restriction medium-treated sample/absorbance of control sample) × 100.

### 2.7. Tube Formation

HRMVECs were seeded at 1000 cells per well in a u-Slide (81506, ibidi, Gräfelfing, Germany) coated with 10 uL basement membrane growth factor reduced matrigel (354230, Corning, Corning, NY, USA). Following a 6 h incubation, resulting tube networks were analyzed by an Olympus BX51 microscope (Olympus Corporation, Tokyo, Japan). The total length of tubule networks and the number of branch points were quantified by ImageJ (Version 1.52) Angiogenesis Analyzer tool.

### 2.8. shRNA Knockdown

The pLKO.1 shRNA vector (Addgene catalog number 10878, Watertown, MA, USA) was utilized to silence the target genes following the manufacturer’s protocol. The oligonucleotides corresponding to the specific genes targeted for silencing are listed. shHRI target sequence (5′-GTACAATGCTTCGTTGTATTT-3′); shPKR target sequence (5′-TCGACCTAACACATCTGAAAT-3′); shPERK target sequence (5′-CGGCAGGTCATTAGTAATTAT-3′); shGCN2 target sequence (5′-CGAGAGATTCTGGATGGATTA-3′); shATF4 target sequence (5′-GCCAAGCACTTCAAACCTCAT-3′); shCTH target sequence (5′-GCACCTCATTATCTTTCATAA-3′).

Lentiviral particles were produced by transfecting HEK293FT cells and subsequently used to infect HRMVECs. To concentrate and purify the virus, the filtered viral supernatant was subjected to centrifugation at 20,000× RPM for 120 min. The resulting supernatant was carefully aspirated, and the viral pellet was resuspended in 1× PBS. Additionally, 20 mL of HEK293T cell supernatant containing the virus was ultracentrifuged and subsequently resuspended in 1 mL of 1× PBS. The resuspended virus was either used immediately or aliquoted into 250 µL portions and stored at −80 °C.

A total of 250 µL of concentrated virus was used to transduce HRMVECs. Following an additional 48 h incubation, transduced cells were selected by the addition of puromycin (1 µg/mL) for a further 48 h.

### 2.9. RNA-Seq

Total RNA was extracted from the tissue using TRIzol^®^ Reagent according to the manufacturer’s instructions. Then RNA quality was determined by 5300 Bioanalyser (Agilent, Santa Clara, CA, USA) and quantified using the ND-2000 (NanoDrop Technologies, Wilmington, DE, USA). Only high-quality RNA sample (OD260/280 = 1.8~2.2, OD260/230 ≥ 2.0, RQN ≥ 6.5, 28S:18S ≥ 1.0, >1 μg) was used to construct the sequencing library.

RNA purification, reverse transcription, library construction, and sequencing were performed in Shanghai Majorbio Bio-pharm Biotechnology Co., Ltd. (Shanghai, China) according to the manufacturer’s instructions. The HRMVECs RNA-seq transcriptome library was prepared following Illumina^®^ Stranded mRNA Prep, Ligation (San Diego, CA, USA) using 1 μg of total RNA. Shortly, messenger RNA was isolated according to the polyA selection method by oligo(dT) beads and then fragmented by fragmentation buffer first. Secondly, double-stranded cDNA was synthesized using a SuperScript double-stranded cDNA synthesis kit (11917020, SuperScript™ Double-Stranded cDNA Synthesis Kit, Invitrogen, Carlsbad, CA, USA) with random hexamer primers. Then the synthesized cDNA was subjected to end-repair, phosphorylation, and adapter addition according to the library construction protocol. Libraries were size selected for cDNA target fragments of 300 bp on 2% Low Range Ultra Agarose followed by PCR amplified using Phusion DNA polymerase (NEB) for 15 PCR cycles. After quantified by Qubit 4.0, the sequencing library was performed on the NovaSeq X Plus platform (PE150) using the NovaSeq Reagent Kit.

The raw paired-end reads were trimmed and quality was controlled by fastp with default parameters. Then clean reads were separately aligned to the reference genome with orientation mode usingHISAT2 (Version 2.2.1) software. The mapped reads of each sample were assembled by StringTie in a reference-based approach. To identify DEGs (differential expression genes) between two different samples, the expression level of each transcript was calculated according to the transcripts per million reads (TPM) method. Functional enrichment analyses including GO and KEGG were performed to identify which DEGs were significantly enriched in GO terms and metabolic pathways at Bonferroni-corrected *p*-value < 0.05 compared with the whole-transcriptome background. GO functional enrichment and KEGG pathway analysis were carried out by Goatools (Version 1.4.4) and Python scipy (Version 1.11.0) software, respectively.

### 2.10. GEO Database

Data Acquisition and Preprocessing Gene expression datasets (GSE234447, GSE211583, GSE233164, and GSE94019) were obtained from the NCBI GEO database (https://www.ncbi.nlm.nih.gov/mesh/, accessed on 11 February 2025). All raw data were subjected to quality control and normalization. Differential expression analysis Differential gene expression analysis was performed using the limma package for R language. After converting the data into an expression matrix, a linear model was fitted using the lmFit function, and empirical Bayesian statistical analysis was performed by the eBayes function. The screening criterion for differentially expressed genes was set as adjusted *p*-value < 0.05, and each gene’s log2 fold change value was calculated. Data visualization was performed by applying the ggplot2 package in R language (Version 4.4.2).

### 2.11. Cell Metabolism Assays

Cellular oxygen consumption was measured using the Seahorse Cell Metabolism Analyzer XF24 (Agilent Technologies). Cells were plated at a density of 10,000 cells and untreated or pretreated with different essential amino acid-restricted human umbilical vein endothelial cell growth medium supplemented with 10% Dialyzed FBS, endothelial cell growth supplement, and heparin. After 16 h, the media was changed to unbuffered XF assay media with 10 mM glucose, 2 mM glutamine, and 1 mM pyruvate at pH 7.4. Oligomycin (1 μM final), RAA (0.5 μM Rotenone + 0.5 μM Antimycin A final), and FCCP (2 μM final) were injected at indicated times according to the manufacturer’s instructions and protocols. All plates were normalized to protein content as measured from the same cells after Seahorse by BCA.

### 2.12. Choroidal-Retinal Flat Mount and Immunostaining

The eyeballs of male C57BL/6J mice were enucleated and immediately fixed in 4% paraformaldehyde for one hour. The RPE-choroid-sclera complex was prepared as a flat mount using four radial incisions and blocked for 1 h with 10% goat serum containing 0.03% Triton X-100. Following extensive washing, the tissue was incubated overnight at 4 °C with isolectin IB4 (1:50). After incubation, the RPE-choroid-sclera complex was washed, flattened, and coverslipped. The CNV area was quantified using ImageJ with optic disc correction applied to the IB4-stained images.

### 2.13. Fundus Fluorescein Angiography (FFA)

One to two weeks following amino acid restriction, the location of CNV leakage was identified using FFA. Real-time FFA images were captured after an intraperitoneal injection of 0.02 mL of a 10% sodium fluorescein solution (Alcon Laboratories, Geneva, Switzerland).

### 2.14. Optical Coherence Tomography (OCT)

OCT images were acquired using spectral-domain optical coherence tomography (OPTO-RIS, OPTOPROBE, Optoprobe Science LTD., Tortola, UK). The long axis of the images was aligned with the plane of the retinal pigment epithelium (RPE), and spindle-shaped hyperreflective areas were identified as CNV lesions. The thickness and length of the lesions were quantified by performing a central scan through the lesion.

### 2.15. Intracellular ROS Production Assays

HRMVECs were seeded in 96-well culture plates (2.0 × 10^3^ cells/well) and grown overnight. After treatment with different essential amino acid-restricted human umbilical vein endothelial cell growth medium supplemented with 10% dialyzed FBS, endothelial cell growth supplement, and heparin for 6 h, the cells were treated as indicated and incubated with fresh medium containing 10 μM DCFH-DA (Sigma-Aldrich) at 37 °C for 30 min. The cells were then washed with PBS 3 times, and fluorescence at 488/525 nm was detected using a multi-mode reader (SpectraMax i3X, Molecular Devices, Shanghai, China). All the data were normalized with cell numbers.

### 2.16. Detection of Mitochondrial Membrane Potential

HRMVECs were seeded in 96-well culture plates (2.0 × 10^3^ cells/well) and grown overnight. After treatment with different essential amino acid-restricted human umbilical vein endothelial cell growth medium supplemented with 10% dialyzed FBS, endothelial cell growth supplement, and heparin for 6 h, the cells were treated as indicated and incubated with fresh medium containing 2 μM JC-1 (meilunbio) at 37 °C for 20 min. The cells were then washed with PBS 3 times, and fluorescence at 490/530 and 525/590 nm was detected using a multi-mode reader (SpectraMax i3X). All the data were normalized with cell numbers.

### 2.17. LC-MS/MS Analysis

Weigh an appropriate amount of amino acid standard and prepare a single standard mother solution with methanol or water. Measure an appropriate amount of each mother liquor to prepare a mixed standard, and dilute it with 10% formic acid methanol-water 1:1 to an appropriate concentration to prepare a working standard solution. The mother liquor and working standard solution were kept refrigerated. Weigh an appropriate amount of the isotope standard (Trp-d3), and prepare the internal standard mother solution with 10% formic acid methanol-water 1:1 to a concentration of 1000 ng/mL. Samples were extracted in 300 μL of 10% formic acid in methanol-water (1:1, *v*/*v*), vortex for 30 s. Put in the adapter of the grinder, immerse it in liquid nitrogen for 5 min, freeze and thaw at room temperature, and shake at 55 Hz for 1 min. Centrifuge at 12,000 rpm and 4 °C for 10 min. Take an appropriate amount of supernatant and add 10% formic acid methanol solution-water (1:1, *v*/*v*) to dilute D times, and vortex for 30 s. Take 100 μL of the supernatant, add 100 μL of 100 ng/mL Trp-d3 internal standard, vortex for 30 s. The supernatant was filtered through 0.22 μm membrane, and the filtrate was added to the LC-MS bottle.

MS Convert software (Proteowizard, v3.0.8789) was applied to convert “.raw” format raw data to “.mzML” format, which would be used for downstream analysis. Extracted-ion chromatograms for each ion were abstracted based on the information of diagnostic ions and quantification ions. Peak areas would be detected by retention times for each compound. The calibration curves were obtained as plots of the peak area ratio of the target compounds to an internal standard versus the target compound concentration. The peak area ratio of target compounds to an internal standard was put into the formula in 2.2 (Experimental Report-Pretreatment) to calculate the concentration in samples. In calculation, all metabolite concentrations less than 0 were reported as not detected (ND).

To assess the technical precision of each experiment, the relative standard deviation of peak areas was calculated for every compound detected in the QC sample (RSD = 100 × standard deviation/average of peak areas) with ideal RSD < 15%.

Only metabolites present in >50% of the samples were kept further analysis. The *t*-test or Mann–Whitney–Wilcoxon test was used to calculate *p*-value. When the comparison group was more than three or more groups, the one-way test of variance or K-H test was used to calculate *p*-value. Differences with *p*-value < 0.05 and VIP > 1 were considered statistically significant (default selection, adjusted according to actual selection).

### 2.18. Statistical Analysis

All data are expressed as the mean ± standard error of the mean (SEM). Significant differences were assessed by unpaired two-tailed Student’s *t*-test, one or two-way analysis of variance (ANOVA) followed by Dunnett’s multiple comparisons test or Bonferroni’s multiple comparisons test where appropriate.

## 3. Results

### 3.1. Essential Amino Acid Restriction Activates Angiogenic Programming in HRMVECs

We carried out a systematic screening to identify deficiency in which EAA was able to regulate angiogenesis. As a result, we found that individual EAA exerted distinct regulatory effects on VEGFA expression and angiogenic function in human retinal microvascular endothelial cells. Preliminary experiments indicated that VEGFA mRNA levels and protein secretion increased significantly between 4 and 24 h, with the optimal time point for detecting vascular formation being 6 h. Consequently, we selected the 6 h time point for gene expression, protein secretion, and vascular formation assays. However, cell count detection became more prominent after 24 h; therefore, the CCK-8 assay was performed at 24 h.

**Figure 1:**
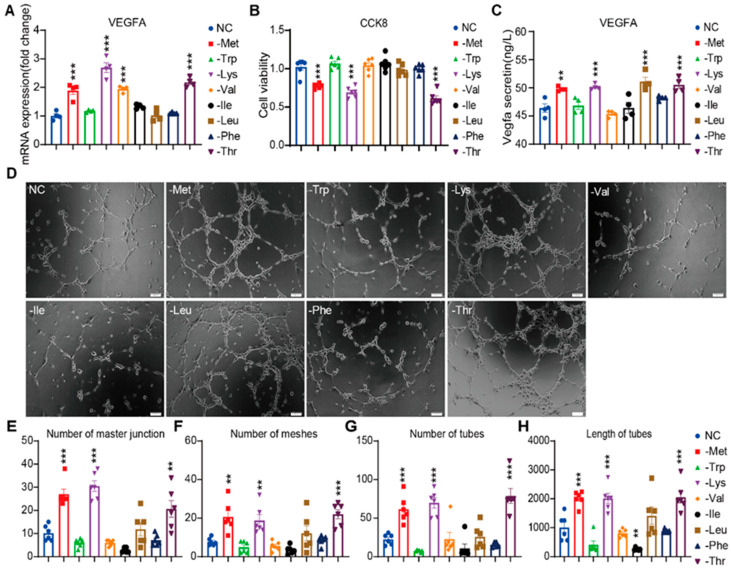
Screening of EAAs restriction to induce endothelial VEGFA expression and angiogenesis. (**A**) VEGFA mRNA levels of HRMVECs cell line cultured in normal control (NC), methionine-deficient (-Met), tryptophan-deficient (-Trp), lysine-deficient (-Lys), valine-deficient (-Val), isoleucine-deficient (-Ile), leucine-deficient (-Leu), isoleucine-deficient (-Ile), phenylalanine-deficient (-Phe) or threonine-deficient (-Thr) media for 6 h (n = 4 replicates). (**B**) The relative proliferation rate of the HRMVECs cell line was measured by CCK-8 assay with the indicated media for 24 h (n = 6 replicates). (**C**) VEGFA secreted protein concentration in the media of HRMVECs cell line incubated in the indicated media for 6 h (n = 4 replicates). (**D**) Tube formation assay: representative capillary-like structures in HRMVECs cell line incubated in the indicated media for 6 h. Scale bar, 100 μm. (**E**–**H**) Quantifying the number of master junctions, meshes, tubes, and the length of tubes in the indicated media for 6 h (n = 6 replicates). Data are represented as means ± SEM. One-way ANOVA with Dunnett’s multiple comparisons test in all analyses. ** *p* < 0.01; *** *p* < 0.001.

Acute (6 h) deficiencies of methionine, lysine, valine, or threonine significantly upregulated VEGFA mRNA levels (1.89-, 2.69-, 1.95-, and 2.18-fold versus complete medium controls, respectively; *p* < 0.001; Figure 1A). In contrast, 24 h deficiency of methionine, lysine, or threonine—but not valine—markedly reduced cell numbers (22%, 31%, and 39% reduction, respectively; *p* < 0.001; Figure 1B). Paralleling transcriptional changes, 6 h methionine, lysine, leucine, or threonine deficiency increased VEGFA protein secretion (*p* < 0.01; Figure 1C), with leucine restriction uniquely induced secretory responses without transcriptional activation. Functional validation through the formation of capillary-like structures demonstrated that 6 h methionine, lysine, or threonine deficiency significantly enhanced angiogenic complexity, increasing junction density, mesh formation, tube number, and total network length versus controls (*p* < 0.001; Figure 1D–H). Leucine restriction showed comparable directional trends that did not reach statistical significance. The conserved methionine-sensing mechanism observed here extended previous reports in human umbilical vein endothelial cells (HUVECs) by demonstrating sulfur-amino acid-specific regulation independent of cysteine availability [7]. The temporal resolution of responses revealed sequential activation—rapid VEGFA (4 h) induction preceding angiogenic remodeling (6 h)—suggesting multistep metabolic reprogramming drives endothelial adaptation to nutrient stress.

### 3.2. Promotion of Angiogenesis by Restriction of EAAs Is Mediated by the eIF2α/ATF4 Axis

The adaptive response of endothelial cells to amino acid limitation involves dynamic crosstalk between nutrient-sensing pathways. Central to this regulation is the GCN2/ATF4 axis, which is activated under amino acid deprivation through GCN2-mediated phosphorylation of eIF2α, driving ATF4-dependent transcriptional programs critical for stress adaptation and metabolic rewiring [7,12]. This pathway enhances the formation of capillary-like structures by upregulating pro-angiogenic mediators such as VEGF. Conversely, mammalian target of rapamycin complex 1 (mTORC1) is a nutrient-responsive hub that drives anabolic processes suppressed during amino acid scarcity, attenuating its pro-angiogenic signaling. However, context-dependent mTORC1 reprogramming under hypoxia or chronic nutrient stress underscores the plasticity of these regulatory networks [13].

**Figure 2:**
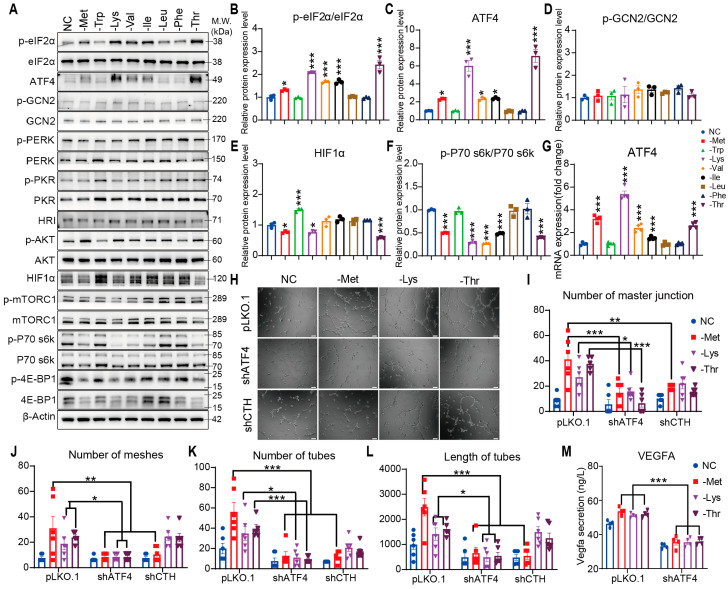
ATF4-dependent endothelial VEGFA expression and angiogenesis under Met, Lys, and Thr restriction. (**A**–**F**) Western blot (**A**) and quantification (**B**–**F**) of eIF2α (p-S51, total), ATF4, GCN2 (p-T899, total), PERK (p-T982, total), PKR (p-T446, total), HRI, AKT (p-S473, total), HIF1α and mTORC1 pathway in HRMVECs cell line cultured in normal control (NC), methionine-deficient (-Met), tryptophan-deficient (-Trp), lysine-deficient (-Lys), valine-deficient (-Val), isoleucine-deficient (-Ile), leucine-deficient (-Leu), isoleucine-deficient (-Ile), phenylalanine-deficient (-Phe) or threonine-deficient (-Thr) media for 6 h (n = 3 replicates). (**G**) Relative ATF4 mRNA expression in HRMVECs cell line incubated in the indicated media for 6 h (n = 4 replicates). (**H**) Tube formation assay: representative capillary-like structures in ATF4 KD, CTH KD, and vector control in HRMVECs cell line incubated in normal control (NC), methionine-deficient (-Met), lysine-deficient (-Lys) or threonine-deficient (-Thr) media for 6 h (n = 4 replicates). Scale bar, 100 μm. (**I**–**L**) Quantifying the number of master junctions, meshes, tubes, and the length of tubes in ATF4 KD, and vector control incubated in the indicated media for 6 h (n = 4 replicates). (**M**) VEGFA secreted protein concentration in the media of HRMVECs cell line in ATF4 KD, and vector control incubated in the indicated media for 6 h (n = 4 replicates). Data are represented as means ± SEM. Asterisks indicate the significance of the difference by one-way ANOVA with Dunnett’s multiple comparisons test or two-way ANOVA with Bonferroni’s multiple comparisons test; * *p* < 0.05, ** *p* < 0.01, *** *p* < 0.001.

Immunoblot analyses revealed that deprivation of methionine, lysine, valine, isoleucine, or threonine robustly enhanced eIF2α phosphorylation and elevated ATF4 expression at both transcriptional and protein levels, while concurrently suppressing mTORC1 activity and hypoxia-inducible factor 1α (HIF1α) accumulation (Figure 2A–G and Supplementary Figure S1A–F). This dissociation from canonical hypoxia-driven angiogenesis mechanisms highlights ATF4 as the predominant mediator of amino acid restriction-induced vascular remodeling. Functional validation using shRNA-mediated knockdown demonstrated that ATF4 and cystathionine gamma-lyase (CTH) are indispensable for methionine restriction-driven capillary morphogenesis, evidenced by suppressed mesh formation, tube elongation, and junctional complexity (Figure 2H,I). In contrast, lysine and threonine restriction retained their pro-angiogenic effects despite CTH depletion, confirming pathway specificity. Notably, all three amino acid deficiencies reduced extracellular VEGFA secretion (Figure 2M), aligning with ATF4’s role in coupling metabolic stress to angiogenic adaptation through the eIF2α/ATF4 axis. These findings establish ATF4 as the central orchestrator of endothelial plasticity under amino acid scarcity, operating independently of mTORC1-HIF1α crosstalk to drive capillary network formation.

### 3.3. HRI Couples Mitochondrial Stress to ATF4-Driven Angiogenesis

**Figure 3:**
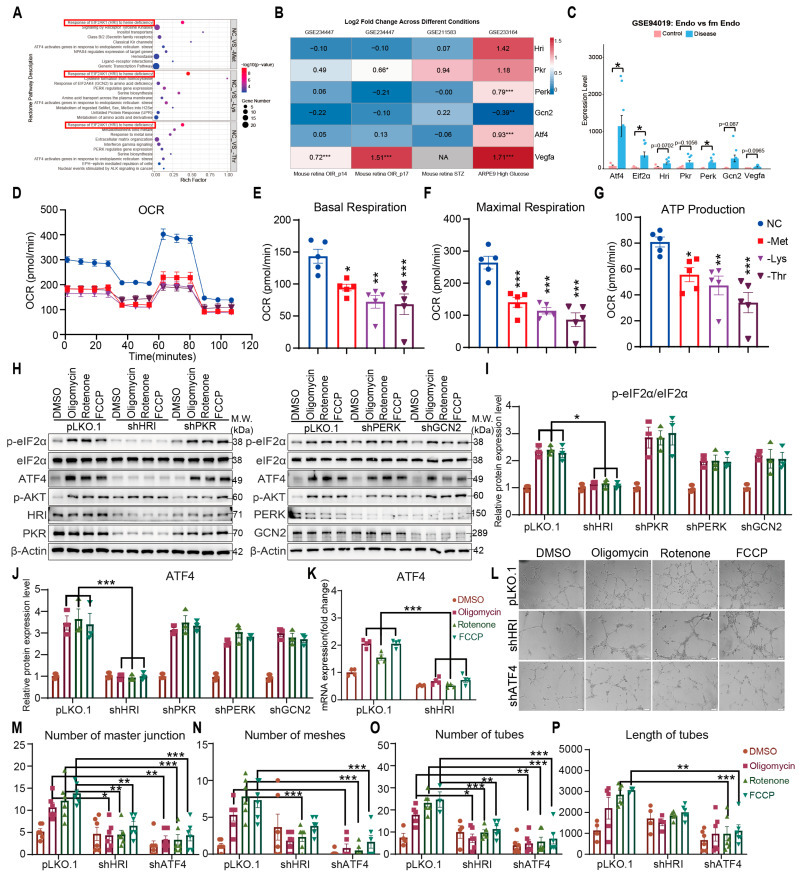
HRI-dependent endothelial VEGFA expression and angiogenesis under electron transport chain inhibition. (**A**) Rectome pathways enrichment analysis of Robust DEGs, and the red box highlights the signal pathways exhibiting consistent alterations. (**B**) Heatmap of DEGs for GSE23447, GSE211583, and GSE233164 datasets. (**C**) The Column Chart Illustrating DEGs in the GSE94019 Dataset. (**D**–**G**) OCR in HRMVECs cell line pretreated for 16 h with normal control (NC), methionine-deficient (-Met), lysine-deficient (-Lys), or threonine-deficient (-Thr) media (n = 5 replicates). OCR (**D**), basal respiration (**E**), maximal respiration (**F**), ATP production (**G**). (**H**–**J**) Western blot (**H**) and quantification (**I**,**J**) of eIF2α (p-S51, total), ATF4, AKT (p-S473), HRI, PKR, PERK, and GCN2 in HRI KD, PKR KD, PERK KD, GCN2 KD, and vector control HRMVECs cell line treated with Oligomycin (1 μmol/L), Rotenone (0.5 μmol/L), and FCCP (2 μmol/L) for 6 h (n = 3 replicates). (**K**) Relative ATF4 mRNA expression in HRMVECs cell line treated with Oligomycin (1 μmol/L), Rotenone (0.5 μmol/L), and FCCP (2 μmol/L) for 6 h (n = 4 replicates). (**L**) Tube formation assay: representative capillary-like structures in HRI KD, PKR KD, PERK KD, GCN2 KD, and vector control HRMVECs cell line treated with Oligomycin (1 μmol/L), Rotenone (0.5 μmol/L), and FCCP (2 μmol/L) for 6 h (n = 4 replicates). Scale bar, 100 μm. (**M**–**P**) Quantifying the number of master junctions, meshes, tubes, and the length of tubes in HRI KD, PKR KD, PERK KD, GCN2 KD, and vector control HRMVECs cell line treated with Oligomycin (1 μmol/L), Rotenone (0.5 μmol/L), and FCCP (2 μmol/L) for 6 h (n = 4 replicates). Data are represented as means ± SEM. Asterisks indicate the significance of the difference by unpaired two-tailed Student’s *t*-test, one-way ANOVA with Dunnett’s multiple comparisons test, or two-way ANOVA with Bonferroni’s multiple comparisons test; * *p* < 0.05, ** *p* < 0.01, *** *p* < 0.001.

To delineate mechanisms linking amino acid restriction to ATF4 activation, bulk RNA sequencing of HRMVECs subjected to 6 h deprivation revealed pronounced HRI pathway enrichment (Figure 3A). Integration of GEO datasets (GSE234447, GSE211583, GSE233164) demonstrated coordinated HRI, ATF4, and VEGFA upregulation in high glucose-exposed ARPE cells (Figure 3B). Integration of these gene expression analyses consistently indicated an activating trend in both the ATF4 signaling pathway and the HRI-mediated stress response pathway under Met/Lys/Thr restriction. This bioinformatic evidence provided a compelling rationale to experimentally interrogate the functional role of HRI in amino acid restriction-induced angiogenesis. In proliferative diabetic retinopathy fibrovascular membranes (GSE94019), HRI elevation in normal versus diabetic endothelial (*p* = 0.0702) paralleled trends in ATF4 and VEGFA (Figure 3C), implicating HRI as a conserved node in metabolic stress responses.

As an eIF2α kinase within the integrated stress response (ISR), HRI bridges mitochondrial dysfunction to translational reprogramming. Under mitochondrial import defects or proteotoxic stress, OMA1-cleaved DELE1 accumulates in the cytosol, directly binding HRI to initiate eIF2α phosphorylation—attenuating global translation while enhancing stress-adaptive transcripts like ATF4 [5,14,15,16]. Amino acid restriction in HRMVECs reduced oxygen consumption rate (OCR), suppressed basal/maximal respiration, and diminished ATP production (Figure 3D–G), indicative of mitochondrial compromise. Pharmacological respiratory chain inhibition (Oligomycin (ATP synthase inhibitor)/Rotenone (Complex I inhibitor)/FCCP (mitochondrial uncoupler), 6 h) induced eIF2α phosphorylation and ATF4 expression effects abolished exclusively by HRI depletion (Figure 3H–K and Supplementary Figure S1K–O). Prolonged mitochondrial inhibition (6 h) enhanced capillary-like structure formation, fully abrogated by HRI or ATF4 knockdown (Figure 3L–P). To further characterize the mitochondrial functional status under amino acid restriction, we assessed additional key parameters. We found that Met/Lys/Thr restriction consistently reduced mitochondrial ROS generation (Figure S1I) and increased the proportion of cells with depolarized mitochondria (loss of ΔΨm) (Figure S1J). Together with the impaired respiratory function, these results provide multifaceted evidence that restriction of these three EAAs induces significant mitochondrial dysfunction, characterized by bioenergetic failure and loss of membrane integrity. Together with the impaired respiratory function, these results provide multifaceted and conclusive evidence that restriction of these three EAAs induces significant mitochondrial dysfunction, characterized by bioenergetic failure and loss of membrane integrity. These findings establish HRI as the primary eIF2α kinase linking amino acid restriction-induced mitochondrial dysfunction to ATF4-driven vascular remodeling, bypassing canonical hypoxia-angiogenesis crosstalk.

### 3.4. Knockdown of HRI or GCN2 Alone Fails to Attenuate EAA Restriction-Driven Angiogenesis

**Figure 4:**
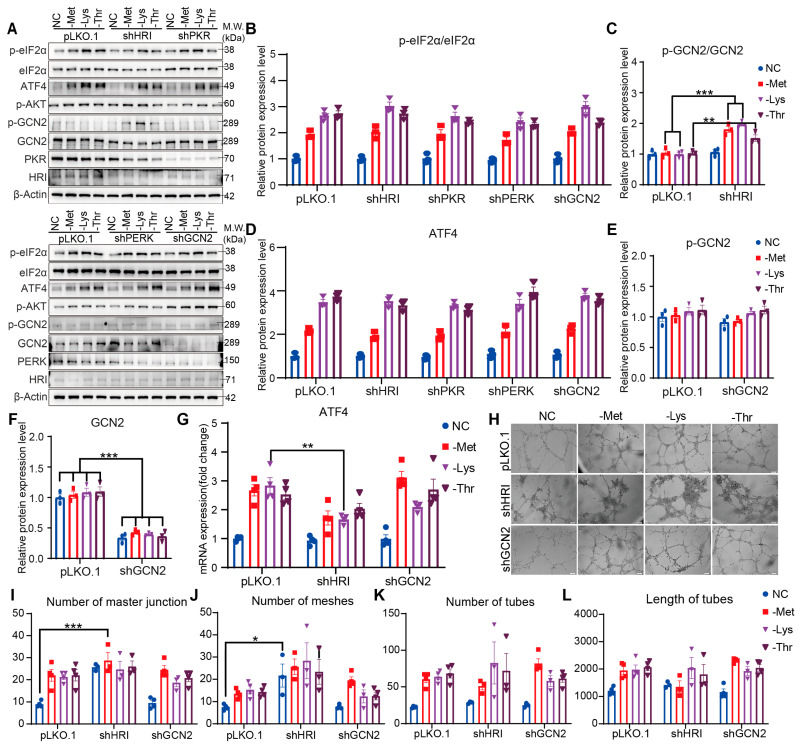
HRI-independent endothelial VEGFA expression and angiogenesis under Met, Lys, and Thr restriction. (**A**) Western blot and quantification (**B**–**F**) of eIF2α (p-S51, total), ATF4, AKT (p-S473), GCN2 (p-T899, total), HRI, PKR, and PERK in HRI KD, PKR KD, PERK KD, GCN2 KD, and vector control HRMVECs cell line incubated in normal control (NC), methionine-deficient (-Met), lysine-deficient (-Lys), or threonine-deficient (-Thr) media for 6 h (n = 3 replicates). (**G**) Relative ATF4 mRNA expression in HRI KD, PKR KD, PERK KD, GCN2 KD, and vector control HRMVECs cell line incubated in normal control (NC), methionine-deficient (-Met), lysine-deficient (-Lys), or threonine-deficient (-Thr) media for 6 h (n = 4 replicates). (**H**) Tube formation assay: representative capillary-like structures in HRI KD, PKR KD, PERK KD, GCN2 KD, and vector control HRMVECs cell line incubated in normal control (NC), methionine-deficient (-Met), lysine-deficient (-Lys), or threonine-deficient (-Thr) media for 6 h (n = 4 replicates). Scale bar, 100 μm. (**I**–**L**) Quantifying the number of master junctions, meshes, tubes, and the length of tubes in HRI KD, PKR KD, PERK KD, GCN2 KD, and vector control HRMVECs cell line incubated in normal control (NC), methionine-deficient (-Met), lysine-deficient (-Lys), or threonine-deficient (-Thr) media for 6 h (n = 4 replicates). Data are represented as means ± SEM. Asterisks indicate the significance of the difference by two-way ANOVA with Bonferroni’s multiple comparisons test; * *p* < 0.05, ** *p* < 0.01, *** *p* < 0.001.

To evaluate the necessity of HRI and GCN2 in amino acid restriction-induced angiogenesis, we performed kinase-specific knockdown experiments. Depletion of HRI or GCN2 kinases in amino acid-deprived HRMVECs did not reduce eIF2α phosphorylation or ATF4 expression and transcription (Figure 4A,B,D,G and Figure S1P–S). Notably, HRI knockdown induced GCN2 activation, suggesting compensatory signaling between these kinases (Figure 4C,E,F). Pro-angiogenic outcomes-including increased vascular proliferation, junction formation, mesh complexity, and tube elongation-remained intact in cells subjected to methionine, lysine, or threonine restriction, even with individual HRI or GCN2 knockdown (Figure 4H,I). These results, combined with the ATF4-dependent angiogenic regulation observed earlier (Figure 2H), indicate that amino acid restriction-driven capillary morphogenesis is resilient to single kinase inactivation. The persistence of angiogenic responses despite individual HRI or GCN2 loss likely reflects functional redundancy between these eIF2α kinases in sustaining ATF4 activation under nutrient stress.

### 3.5. HRI and GCN2 Cooperatively Drive ATF4-Dependent Angiogenesis Through eIF2α Phosphorylation Under EAA Restriction

**Figure 5:**
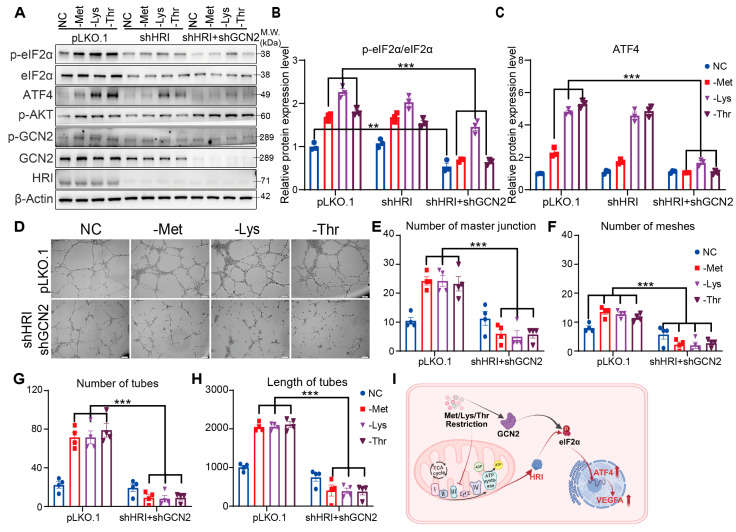
Met, Lys, and Thr restriction modulates ATF4 expression via HRI and GCN2, consequently influencing angiogenesis. (**A**) Western blot and quantification (**B**,**C**) of eIF2α (p-S51, total), ATF4, AKT (p-S473), GCN2 (p-T899, total), and HRI in HRI KD, HRI+GCN2 DKD, and vector control HRMVECs cell line incubated in normal control (NC), methionine-deficient (-Met), lysine-deficient (-Lys), or threonine-deficient (-Thr) media for 6 h (n = 3 replicates). (**D**) Tube formation assay: representative capillary-like structures in HRI+GCN2 DKD, and vector control HRMVECs cell line incubated in normal control (NC), methionine-deficient (-Met), lysine-deficient (-Lys), or threonine-deficient (-Thr) media for 6 h (n = 4 replicates). Scale bar, 100 μm. (**E**–**H**) Quantifying the number of master junctions, meshes, tubes, and the length of tubes in HRI+GCN2 DKD, and vector control HRMVECs cell line incubated in normal control (NC), methionine-deficient (-Met), lysine-deficient (-Lys), or threonine-deficient (-Thr) media for 6 h (n = 6 replicates). (**I**) Model for Regulation of Angiogenesis by Met, Lys, and Thr Restriction. Data are represented as means ± SEM. Asterisks indicate the significance of the difference by two-way ANOVA with Bonferroni’s multiple comparisons test; ** *p* < 0.01, *** *p* < 0.001.

Building on the observed redundancy between HRI and GCN2, we next interrogated their synergistic roles by generating HRMVECs with combined HRI/GCN2 knockdown. Immunoblot analysis revealed that dual kinase depletion, unlike single knockdowns, significantly attenuated eIF2α phosphorylation and reduced ATF4 protein levels following 6 h methionine, lysine, or threonine restriction (Figure 5A–C and Figure S2A–D). Prolonged amino acid deprivation (6 h) in double-knockout cells markedly suppressed capillary morphogenesis, with pronounced reductions in junction density, mesh complexity, tube number, and network length (Figure 5D–H). These data demonstrate that HRI and GCN2 act cooperatively to amplify eIF2α/ATF4 signaling under amino acid scarcity. Mechanistically, amino acid restriction engages both mitochondrial stress-mediated HRI activation and direct GCN2 sensing, converging on ATF4 to drive VEGFA-dependent angiogenic adaptation (Figure 5I). This kinase synergy ensures robust vascular remodeling despite transient or compartment-specific nutrient challenges.

### 3.6. Dietary Restriction of Methionine, Lysine, and Threonine Exacerbates Choroidal Neovascularization in a Laser-Induced AMD Mouse Model

The laser-induced choroidal neovascularization model has proven indispensable for elucidating molecular mechanisms in age-related macular degeneration, particularly in defining the role of signaling pathways such as VEGF in promoting the formation of capillary-like structures. Studies leveraging this model have established VEGF pathway activation as a critical driver of pathological angiogenesis, with therapeutic VEGF inhibition effectively reducing CNV severity and retinal damage [17,18]. Beyond pharmacological targets, this model has enabled the evaluation of dietary interventions, including omega-3 fatty acids, which attenuate inflammatory responses and CNV progression [19,20]. Additionally, this system has rigorously tested emerging anti-complement and anti-VEGF therapies, underscoring its translational utility [21,22].

**Figure 6:**
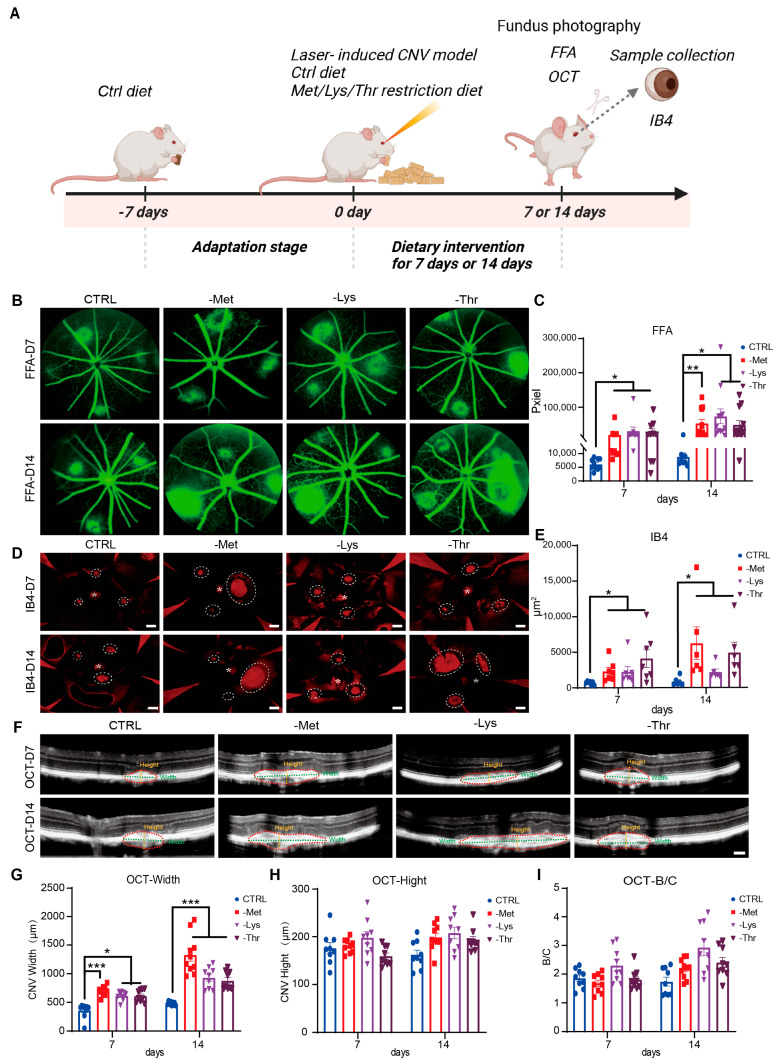
Dietary restriction of methionine, lysine, and threonine exacerbates choroidal neovascularization in a laser-induced AMD mouse model. (**A**) Experimental timeline and schematics. Retinal laser-induced photocoagulation (3 spots per eye) and Met, Lys, and Thr-restricted diet initiated on day 0. On days 7 (n = 6 replicates) and 14 (n = 6 replicates), fundus photography, FFA (n = 6 replicates), IB4 (n = 3 replicates), and OCT (n = 6 replicates) were conducted to assess the influence of diet. (**B**) Representative FFA images in the mouse before and after the treatment. (**C**) Quantitative analysis of relative FFA areas 7 and 14 days after treatment with all groups. (**D**) Images obtained on days 7 and 14 of laser-induced CNV mice treated with Met, Lys, and Thr-restricted diet. The position of the CNV area (white dotted circle) and optic disc (asterisk) is depicted in the fluorescence images of choroid flat mounts stained with IB4 (red). Scale bar, 50 μm. (**E**) Quantitative analysis of relative CNV areas 7 and 14 days later after treatment with all groups. (**F**) The largest horizontal diameter of a CNV served as the definition of CNV width. The distance between the highest point of the CNV and Bruch’s membrane was used to calculate CNV height. Scale bar, 200 μm. (**G**–**I**) Quantitative analysis of CNV widths (**G**), heights (**H**), and B/C ratios (**I**) 7 and 14 days later after treatment with all groups. Data are represented as means ± SEM. Asterisks indicate the significance of the difference by one-way ANOVA with Dunnett’s multiple comparisons test; * *p* < 0.05, ** *p* < 0.01, *** *p* < 0.001.

To investigate the impact of methionine, lysine, and threonine restriction on choroidal vascular pathology, we employed this validated laser-induced AMD model in mice, maintaining amino acid levels at one-quarter of control diets to prevent malnutrition (Figure 6A). Longitudinal analyses via FFA and OCT revealed progressive exacerbation of CNV in amino acid-restricted cohorts. FFA imaging demonstrated enlarged lesion leakage areas in methionine-, lysine-, and threonine-deficient groups at both 7- and 14-day endpoints, whereas control cohorts exhibited stable leakage profiles (Figure 6B,C). Choroidal spread analyses corroborated these findings, with IB4 staining (a vascular marker) showing expanded neovascular territories in intervention groups compared to controls (Figure 6D,E). OCT quantification further revealed augmented CNV thickness in amino acid-restricted mice, though no significant alterations in width or choroidal layer-to-neovascular membrane ratios were observed (Figure 6F–I). These data collectively demonstrate that dietary limitation of methionine, lysine, and threonine potentiates laser-induced formation of capillary-like structures in the choroid, implicating amino acid availability as a modulator of pathological angiogenesis in AMD progression.

## 4. Discussion

Although recent research has linked certain amino acids—such as glutamine [23,24], arginine [25,26], those with sulfur [7], glycine [27], and leucine [28]—to the process of new blood vessel formation, the broader influence of amino acids on reshaping blood vessels is not yet clear. In comparison to prior work, such as Longchamp et al. (2018) who demonstrated that sulfur amino acid restriction (primarily methionine) triggers angiogenesis via GCN2/ATF4-mediated VEGF and H2S production in endothelial cells, our study extends this by identifying lysine and threonine as additional potent inducers that operate independently of the CTH-H2S pathway, potentially through direct ATF4-VEGFA promoter interactions [7]. This specificity for methionine, lysine, and threonine may arise from their unique roles in depleting charged tRNAs and activating nutrient-sensing kinases like GCN2 and HRI, unlike branched-chain amino acids (e.g., leucine), which primarily signal via mTOR without robust ATF4 induction, as evidenced in endothelial biology reviews [29]. Furthermore, studies on ATF4 regulation under metabolic stress, such as in lipid overload models, align with our findings by showing ATF4’s central role in angiogenic adaptation, but our work highlights EAA-specific triggers in retinal contexts [30]. For human extrapolation, clinical associations link low levels of lysine, methionine, and threonine to metabolic imbalances and immune dysfunction, suggesting potential relevance to AMD progression in aging populations with subclinical deficiencies [31]; additionally, methionine metabolism’s influence on glioma angiogenesis via CXCL8 under restriction implies broader therapeutic implications for vascular diseases, including dietary interventions to modulate these EAAs in humans [32]. Through systematic functional screening of eight essential amino acid restrictions, we identify methionine, lysine, and threonine as potent inducers of ATF4 expression (Figure 2), VEGFA secretion, and angiogenic activation (Figure 1). In our approach, we screened limitations in eight key amino acids that must be obtained from diet, revealing that shortages in methionine, lysine, and threonine strongly boost ATF4 levels (Figure 2), increase VEGFA release, and trigger vessel-forming activity (Figure 1). Mechanistically, compensatory crosstalk between HRI and GCN2 maintains eIF2α phosphorylation and ATF4 signaling during single kinase inhibition, whereas dual inactivation disrupts both eIF2α/ATF4 signaling and capillary morphogenesis. In a laser-induced choroidal neovascularization model, dietary restriction of Met, Lys, or Thr exacerbates pathological angiogenesis, positioning amino acid availability as a tunable metabolic checkpoint in retinal vascular plasticity.

Notably, while Met restriction aligns with reported ATF4-CTH-H2S-mediated angiogenesis [7,33,34], Lys and Thr restriction operate independently of CTH, suggesting divergent downstream mechanisms despite shared ATF4 activation. This distinction is critical given CTH’s role in catalyzing H2S production gasotransmitter promoting vasodilation and angiogenesis via VEGFA induction [35]. Intriguingly, ATF4 directly binds the VEGFA promoter to enhance transcription during ER stress [36,37], a pathway co-opted in tumor angiogenesis [38,39]. Our findings imply that Lys/Thr restriction may bypass H2S-dependent signaling to engage ATF4-VEGFA crosstalk directly.

The PERK/eIF2α/ATF4 axis, known to regulate VEGFA secretion under glucose deprivation [40,41], exhibits context-dependent functionality: leucine restriction promotes VEGFA secretion without altering mRNA levels or ATF4 expression, suggesting post-transcriptional regulation. Angiogenic outcomes likely integrate multiple growth factors (e.g., FGF, PDGF, angiopoietins [42,43]), potentially explaining why valine/isoleucine restriction-despite elevating ATF4-requires synergistic inputs to drive vascular proliferation.

Although ATF4 stabilizes HIF1α under hypoxia [40,44] and mTORC1 regulates ATF4 turnover [45], both pathways are suppressed during Met/Lys/Thr restriction-induced angiogenesis (Figure 2), indicating mechanistic uncoupling from canonical hypoxia or nutrient-sensing networks. This establishes amino acid scarcity as a distinct regulatory paradigm in endothelial adaptation. Building on international literature, our results contrast with broader amino acid roles in tumor angiogenesis, where glutamine and arginine drive vessel formation via metabolic fueling [23,25], but align with EAA restriction studies showing pro-angiogenic effects in non-ocular models [28,29]. The particular efficacy of methionine, lysine, and threonine likely stems from their involvement in one-carbon metabolism and stress signaling, differentiating them from other EAAs like valine, which showed transient effects in our screening [30]. Regarding human relevance, epidemiological data associate EAA imbalances with age-related diseases, including AMD, where dietary methionine restriction in rodents extends lifespan and reduces inflammation, potentially translatable to human precision nutrition strategies for preventing pathological angiogenesis [31,32].

The significant restriction of methionine, lysine, and threonine was indeed designed as an experimental proof-of-concept to robustly activate and characterize the proposed nutrient-sensing pathways, rather than to mimic a common human dietary deficiency. It is true that severe deficiencies of these particular essential amino acids (EAAs) are uncommon in typical human diets. However, our new metabolic data (Supplementary Figure S2E–G) revealed a fascinating phenomenon: despite the dietary restriction, serum levels of the three targeted EAAs remained unchanged after one week. This suggests the existence of potent systemic adaptive mechanisms that work to maintain circulating EAA homeostasis, a finding consistent with other studies of dietary amino acid challenges [46,47]. This metabolic compensation likely involves mobilization of tissue stores and prioritization of protein conservation, implying that the physiological impact of our dietary intervention may be more accurately reflected by the activation of cellular nutrient-sensing pathways (like GCN2) in local tissues rather than by static serum measurements. This indicates that the one-week dietary restriction protocol, while sufficient to activate a pro-angiogenic signaling axis locally, was well-tolerated systemically and did not induce overt metabolic dysfunction in the mice.

The core implication of our findings is not that humans commonly experience such extreme deficiencies, but rather that even when the body successfully buffers systemic EAA levels, localized metabolic sensing of amino acid availability—potentially at the level of the endothelium or within specific tissue microenvironments—can exert powerful effects on vascular pathophysiology. This concept is supported by emerging research highlighting the role of local nutrient sensing in disease [48]. Furthermore, milder, chronic insufficiencies of these EAAs could occur in human populations due to malnutrition, specific dietary patterns, or malabsorption syndromes, and our findings highlight a potential vascular consequence of such states.

From a therapeutic perspective, our work underscores the potential of targeting the identified HRI/GCN2-ATF4 axis pharmacologically, rather than through extreme dietary manipulation, to achieve a controlled modulation of pathological angiogenesis. This could offer a more viable and translatable strategy for managing conditions like neovascular AMD.

Our experimental focus on the dual knockdown of HRI and GCN2, beyond addressing potential kinase redundancy, was principally guided by a mechanistic hypothesis. The core trigger of our model is essential amino acid restriction, for which GCN2 serves as the canonical sensor. Concurrently, our data revealed that this restriction induces mitochondrial dysfunction (Figure 3D–G), and HRI is the established primary sensor for mitochondrial proteotoxic stress. We therefore postulated that the HRI/GCN2 combination represents a biologically coherent convergent model: GCN2 directly sensing the initial amino acid deficit and HRI responding to the subsequent mitochondrial distress, thus providing a synergistic, two-hit activation of the integrated stress response. This rationale is further supported by our gene expression analyses, which specifically pointed to a significant enrichment of the HRI pathway under these conditions (Figure 3A). While functional redundancy with other eIF2α kinases remains a formal possibility, the lack of compensatory activation of PKR or PERK upon HRI or GCN2 knockdown (Figure 4) suggests their involvement in this specific metabolic stress axis may be secondary.

Amino acid restriction activates the GCN2/eIF2α pathway through mechanisms such as mTOR-mediated allosteric regulation, AhR-dependent transcriptional reprogramming, and protein complex formation, without altering GCN2 phosphorylation levels [49,50,51]. Restriction of methionine, lysine, and threonine specifically did not increase GCN2 phosphorylation (p-T899). This suggests activation may occur via alternative GCN2 phosphorylation sites or involve mechanisms maintaining constitutive GCN2 phosphorylation. The precise molecular basis requires further investigation. Our findings on the HRI and GCN2 double knockdown demonstrate compensatory action within the integrated stress response (ISR) network, where single kinase inhibition fails to abolish eIF2α/ATF4 signaling, but dual inactivation disrupts it, as supported by studies on ISR kinase redundancy [15,16,49,50,51]. This compensation ensures robust adaptation to nutrient stress, aligning with our data showing abolished angiogenic responses only in double knockouts.

Mitochondrial stress may further potentiate angiogenesis through ROS-mediated VEGFA activation [14,52] and ISR engagement. Beyond HRI/GCN2-eIF2α-ATF4 signaling, Met/Lys/Thr restriction could thus exploit mitochondrial retrograde signaling to amplify angiogenic responses, highlighting metabolic-inflammatory crosstalk in vascular remodeling.

A few constraints should be noted: (1) Even though joint activation of HRI and GCN2 on eIF2α leads to ATF4 and VEGFA promoting cell growth in retinal endothelial cells, other unknown factors might also play a role; (2) How well this pathway applies to real-world AMD cases or other blood vessel diseases needs further confirmation; (3) The exact way HRI and GCN2 interact at the phosphorylation level is still unclear. Ongoing studies employing endothelial-specific HRI/GCN2 knockout models will clarify their roles in choroidal neovascularization and broader vascular pathologies.

## 5. Conclusions

Collectively, our study demonstrates that systemic essential amino acid restriction modulates endothelial angiogenic activity through stress-responsive signaling pathways. This nutrient-sensitive mechanism operates independently of canonical hypoxia-HIF1α or mTORC1 signaling, instead engaging mitochondrial stress-triggered HRI activation and GCN2-mediated amino acid sensing. These pathways converge on eIF2α phosphorylation to enhance ATF4-dependent metabolic adaptation, which we propose contributes to pathological vascular remodeling. These findings suggest that fine-tuning amino acid availability or selectively targeting components of the HRI/GCN2-eIF2α-ATF4 axis may offer therapeutic opportunities for angiogenesis-related disorders.

Looking forward, our findings open several avenues for future research. First, generating endothelial-specific HRI/GCN2 double knockout mouse models will be essential to definitively establish the cell-autonomous role of this pathway in pathological angiogenesis in vivo. Second, high-throughput screening for specific pharmacological inhibitors or activators of HRI and GCN2 kinases could yield novel therapeutic candidates. Third, exploring whether milder, more clinically relevant dietary interventions (e.g., periodic restriction or specific amino acid imbalances) can modulate this pathway warrants further investigation.

From a practical standpoint, this work moves beyond the counterintuitive strategy of severe dietary restriction and instead highlights the HRI/GCN2-eIF2α-ATF4 axis as a promising new signaling network for therapeutic intervention. Our data provide a strong rationale for developing small-molecule compounds that target this pathway to achieve a controlled anti-angiogenic effect. Furthermore, these findings contribute to the growing field of precision nutrition by identifying specific amino acids whose dietary modulation could potentially influence vascular health, paving the way for more targeted nutritional strategies for populations at risk of neovascular diseases.

## Data Availability

The original contributions presented in this study are included in the article and Supplementary Materials. Further inquiries can be directed to the corresponding authors.

## Supplementary Materials

The following supporting information can be downloaded at: https://www.mdpi.com/article/10.3390/nu17183006/s1, Table S1: Food table; Figure S1: Supplementary quantitative experimental figure; Figure S2: Serum amino acid metabolome.

## Abbreviations

The following abbreviations are used in this manuscript:

AA	amino acid
EAA	essential amino acid
AMD	age-related macular degeneration
VEGF-A	vascular endothelial growth factor A
HRMVECs	human retinal microvascular endothelial cells
HRI	heme-regulated inhibitor
PKR	protein kinase RNA-activated
PERK	protein kinase R-like endoplasmic reticulum kinase
GCN2	general control nonderepressible 2
ATF4	activating transcription factor 4
CNV	choroidal neovascularization
GSH	glutathione
OXPHOS	oxidative phosphorylation
ROS	reactive oxygen species
eIF2α	eukaryotic translation initiation factor 2α
SIBS	Shanghai Institute for Biological Sciences
CAS	Chinese Academy of Sciences
AL	ad libitum
M	methionine
K	lysine
T	threonine
ECM	endothelial cell medium
ECGS	endothelial cell growth supplement
FBS	fetal bovine serum
qPCR	quantitative PCR
DEG	differential expression genes
TPM	transcripts per million reads
FFA	fundus fluorescein angiography
OCT	optical coherence tomography
RPE	retinal pigment epithelium
SEM	standard error of the mean
ANOVA	analysis of variance
HUVECs	human umbilical vein endothelial cells
mTORC1	mechanistic target of rapamycin complex 1
HIF1α	hypoxia-inducible factor 1α
CTH	cystathionine gamma-lyase
ISR	integrated stress response
OCR	oxygen consumption rate
