# Supplementary material for: Identification of Amino Acids That Regulate Angiogenesis and Alter Pathogenesis of a Mouse Model of Choroidal Neovascularization
Source: Nutrients. 2025 Sep 19;17(18):3006. doi: 10.3390/nu17183006 (PMC12472552; doi:10.3390/nu17183006)
Supplement: Supplementary file 1 [file nutrients-17-03006-s001.zip › nutrients-3813593-supplementary.pdf]

Supplemental Information

Identification of amino acids that regulate angiogenesis and alter pathogenesis of a mouse model of choroidal neovascularization

Chenchen Li, Jiawen Wu, Yingke Zhao, Jing Zhu, Xinyu Zhu, Yan Chen, and Jihong Wu

	Ctrl diet (FB-A10021B)		-Met diet (1.5g/kg)		-Lys diet (3.5g/kg)		-Thr (2g/kg)	
Ingredients	Quality (g)	Energy (kcal)	Quality (g)	Energy (kcal)	Quality (g)	Energy (kcal)	Quality (g)	Energy (kcal)
L-Arginine	10	40	10	40	10	40	10	40
L-Histidine-HCl-H_2O	6	24	6	24	6	24	6	24
L-Isoleucine	8	32	8	32	8	32	8	32
L-Leucine	12	48	12	48	12	48	12	48
L-Lysine-HCl	14	56	14	56	3.5	14	14	56
L-Methionine	6	24	1.5	6	6	24	6	24
L-Phenylalanine	8	32	8	32	8	32	8	32
L-Threonine	8	32	8	32	8	32	2	8
L-Tryptophan	2	8	2	8	2	8	2	8
L-Valine	8	32	8	32	8	32	8	32
L-Alanine	10	40	10	40	10	40	10	40
L-Asparagine-H_2O	5	20	5	20	5	20	5	20

L-Aspartate	10	40	10	40	10	40	10	40
L-Cystine	4	16	4	16	4	16	4	16
L-Glutamic Acid	30	120	30	120	30	120	30	120
L-Glutamine	5	20	5	20	5	20	5	20
Glycine	10	40	10	40	10	40	10	40
L-Proline	5	20	5	20	5	20	5	20
L-Serine	5	20	5	20	5	20	5	20
L-Tyrosine	4	16	4	16	4	16	4	16
Corn Starch	550.5	2202	555	2220	561	2244	556.5	2226
Maltodextrin	125	500	125	500	125	500	125	500
Cellulose	50		50		50		50	
Corn Oil	50	450	50	450	50	450	50	450
Mineral Mix	35		35		35		35	
Sodium Bicarbonate	7.5		7.5		7.5		7.5	

Vitamin Mix	10	40	10	40	10	40	10	40
Choline Bitrartrate	2		2		2		2	
Yellow	0		0		0		0.05	
Blue	0		0		0.05		0	
Red	0		0.05		0		0	
**Total**	1000	3872	1000.05	3872	1000.1	3872	1000.1	3872
	Quality ratio	Energy ratio	Quality ratio	Energy ratio	Quality ratio	Energy ratio	Quality ratio	Energy ratio
Protein	0.1700	0.1756	0.1655	0.1710	0.1595	0.1648	0.1640	0.1694
Fat	0.0500	0.1162	0.0500	0.1162	0.0500	0.1162	0.0500	0.1162
Carbohydrate	0.6855	0.7082	0.6900	0.7128	0.6960	0.7190	0.6915	0.7144

Table S1: Food table.

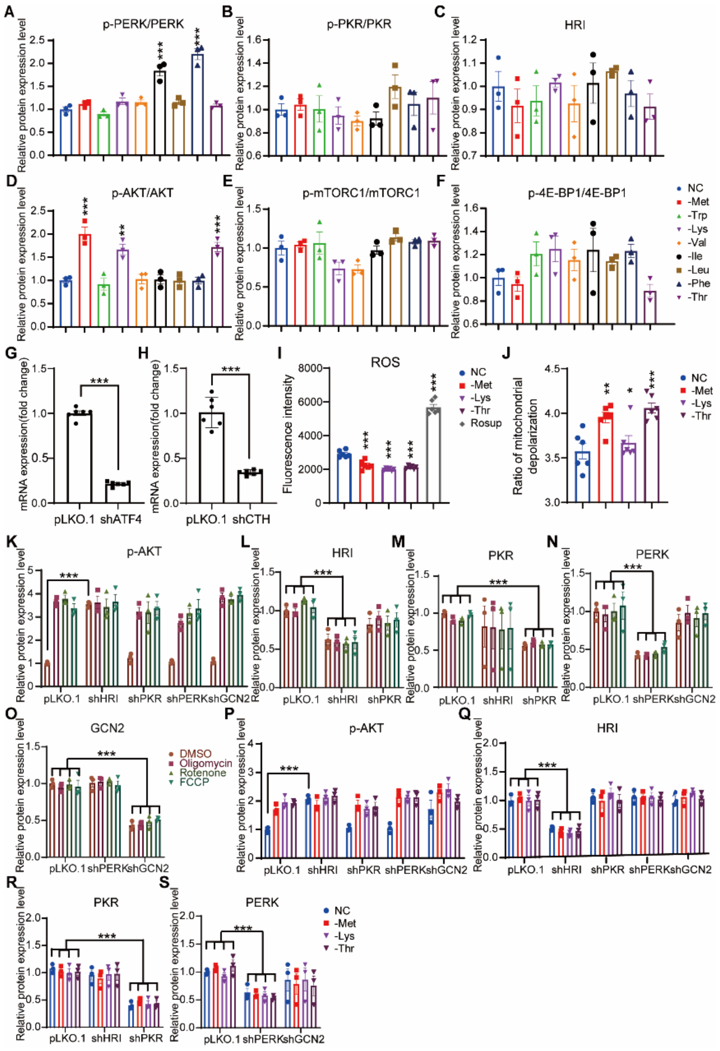

Figure S1: Supplementary quantitative experimental figure. (A–F) Quantification of p-PERK(p-T982)/PERK, p-PKR(p-T446)/PKR, HRI,

p-AKT (p-S473)/AKT, p-mTORC1(p-S2448)/mTORC1 and p-4E-BP1(p-T37/46)/4E-BP1 pathway in HRMVECs cell line cultured in normal control (NC), methionine-deficient (-Met), tryptophan-deficient (-Trp), lysine-deficient (-Lys), valine-deficient (-Val), isoleucine-deficient (-Ile), leucine-deficient (-Leu), isoleucine-deficient (-Ile), phenylalanine-deficient (-Phe) or threonine-deficient (-Thr) media for 6 h (n = 3 replicates). (**G-H**) Relative ATF4 and CTH mRNA expression in HRMVECs cell line (n = 6 replicates). (**I**) The generation of ROS in HRMVECs cell line incubated in normal control (NC), methionine-deficient (-Met), lysine-deficient (-Lys) or threonine-deficient (-Thr) media for 6 h (n = 6 replicates). Rosup (1 μM final). (**J**) The ratio of mitochondrial depolarization in HRMVECs cell line incubated in normal control (NC), methionine-deficient (-Met), lysine-deficient (-Lys) or threonine-deficient (-Thr) media for 6 h (n = 6 replicates). (**K–O**) Quantification of p-AKT (p-S473), HRI, PKR, PERK, and GCN2 in HRI KD, PKR KD, PERK KD, GCN2 KD, and vector control HRMVECs cell line treated with Oligomycin (1 μmol/L), Rotenone (0.5 μmol/L), and FCCP (2 μmol/L) for 6 h (n = 3 replicates). (**P-S**) Quantification of p-AKT (p-S473), HRI, PKR, and PERK in HRI KD, PKR KD, PERK KD, GCN2 KD, and vector control HRMVECs cell line incubated in normal control (NC), methionine-deficient (-Met), lysine-deficient (-Lys), or threonine-deficient (-Thr) media for 6 h (n = 3 replicates). Asterisks indicate the significance of the difference by one-way ANOVA with Dunnett's multiple comparisons test or two-way ANOVA with Bonferroni's multiple comparisons test; * $p < 0.05$, ** $p < 0.01$, *** $p < 0.001$.

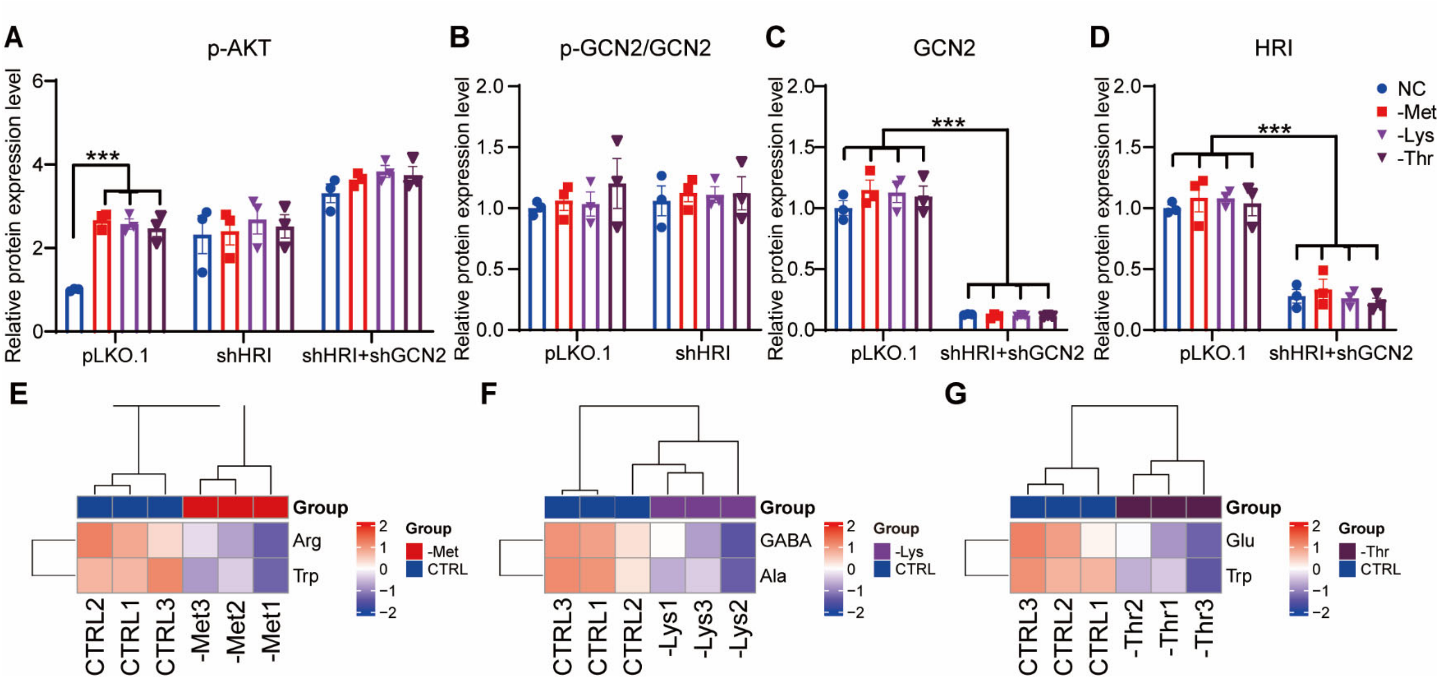

Figure S2: Serum amino acid metabolome. (**A**–**D**) Quantification of p-AKT (p-S473), p-GCN2 (p-T899)/GCN2, GCN2, and HRI in HRI KD, HRI+GCN2 DKD, and vector control HRMVECs cell line incubated in normal control (NC), methionine-deficient (-Met), lysine-deficient (-Lys), or threonine-deficient (-Thr) media for 6 h (n = 3 replicates). (**E-G**) Quantitative analysis of amino acid content in serum 7 days after treatment with Met, Lys, and Thr-restricted diet (n = 3 replicates). Asterisks indicate the significance of the difference by one-way ANOVA with Dunnett's multiple comparisons test or two-way ANOVA with Bonferroni's multiple comparisons test; *** $p < 0.001$.
